# Supplementary material for: Liquid–liquid phase separation of LARP7 restrains HIV-1 replication
Source: EMBO Rep. 2025 Mar 20;26(8):1935–56. doi: 10.1038/s44319-025-00421-9 (PMC12019422; doi:10.1038/s44319-025-00421-9)
Supplement: Supplementary file 7 — Expanded View Figures [file 44319_2025_421_MOESM7_ESM.pdf]

Expanded View Figures

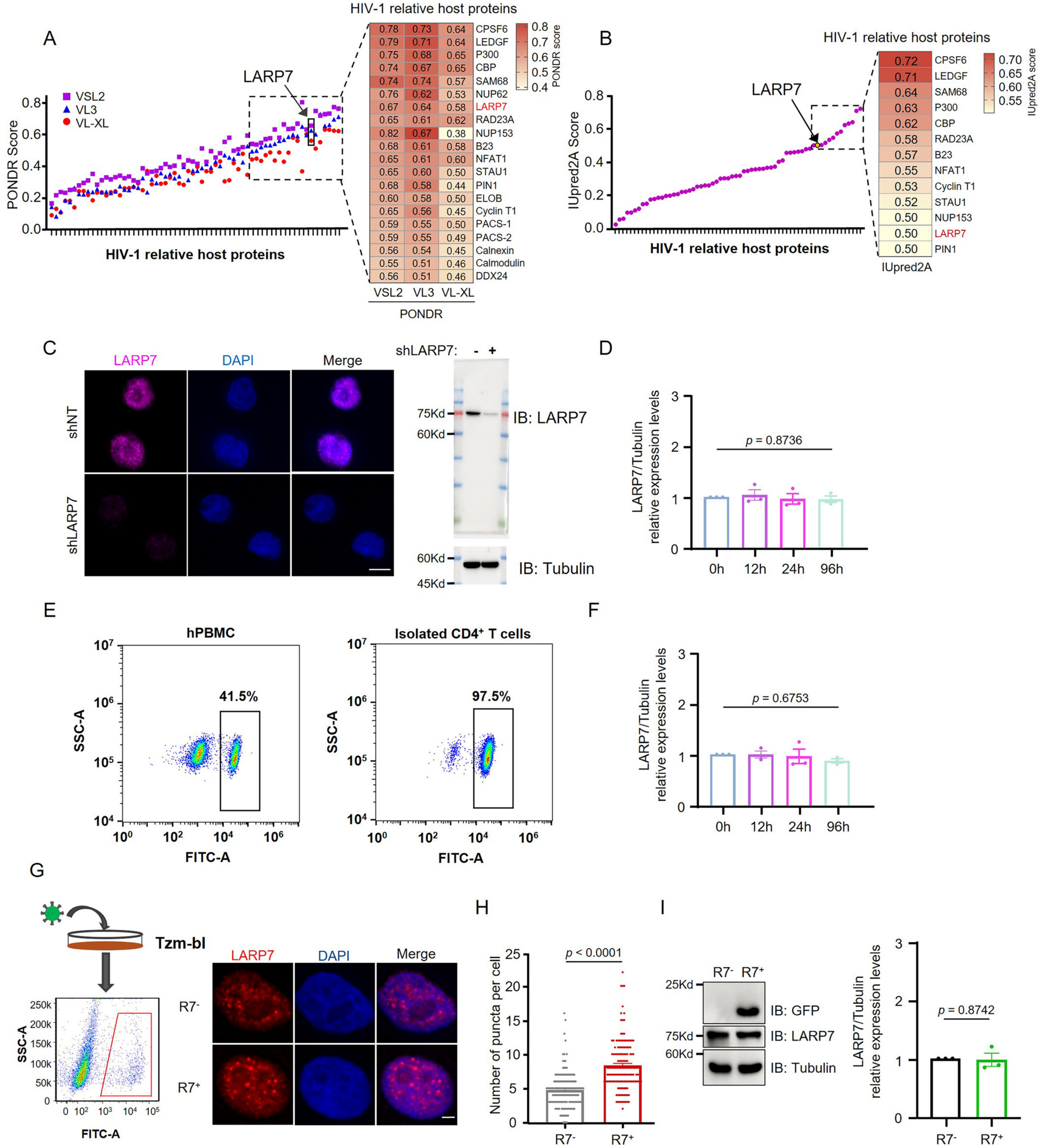

 © The Author(s)

Figure EV1. LARP7 undergoes liquid-liquid phase separation after HIV-1 infection.

(**A**) IDR content scoring and ranking of host proteins reported to be involved in HIV-1 replication using the predictor PONDR. (**B**) IDR content scoring and ranking of host proteins were reported to be involved in HIV-1 replication using the predictor IUPred2A. (**C**) Western blotting and immunofluorescence analyses (Scale bar, 5 μm) for Jurkat cells, testing the specificity of the anti-LARP7 antibody. (**D**) Quantification of the relative expression level of LARP7 and tubulin in Jurkat cells at different times after HIV-1 R7-ΔEnv-GFP infection. The western blotting results were analyzed by using ImageJ software. Each data point represents an independent biological replicate ($n = 3$). One-way analysis of variance (ANOVA) was used for statistical analysis, and exact p values are represented in the figure, mean ± SEM. (**E**) FACS analysis of the percentage of primary $CD4^+$ T cells isolated from human primary peripheral blood mononuclear cells of healthy individuals. (**F**) Quantification of the relative expression level of LARP7 and tubulin in primary $CD4^+$ T cells at different times after HIV-1 R7-ΔEnv-GFP infection. The western blotting results were analyzed by using ImageJ software. Each data point represents an independent biological replicate ($n = 3$). One-way analysis of variance (ANOVA) was used for statistical analysis, and exact p values are represented in the figure, mean ± SEM. (**G**) Left, flow cytometry-sorted GFP-positive and GFP-negative Tzm-bl cells 24 h after HIV-1 R7-ΔEnv-GFP infection. Right, immunofluorescence of LARP7 in sorted cells. Scale bar, 2 μm. (**H**) Quantification of the number of puncta in individual cells from Fig. EV1G; 150 cells were analyzed in each group. A two-tailed unpaired student's t-test was used for statistical analysis, and exact p values are represented in the figure, mean ± SEM. (**I**) Left, western blotting analysis of LARP7 expression in sorted Tzm-bl cells from Fig. EV1G. Right, quantification of the relative expression level of LARP7 and tubulin. The western blotting results were analyzed by using ImageJ software. Each data point represents an independent biological replicate ($n = 3$). A two-tailed unpaired student's t-test was used for statistical analysis, and exact p values are represented in the figure, mean ± SEM.

© The Author(s)

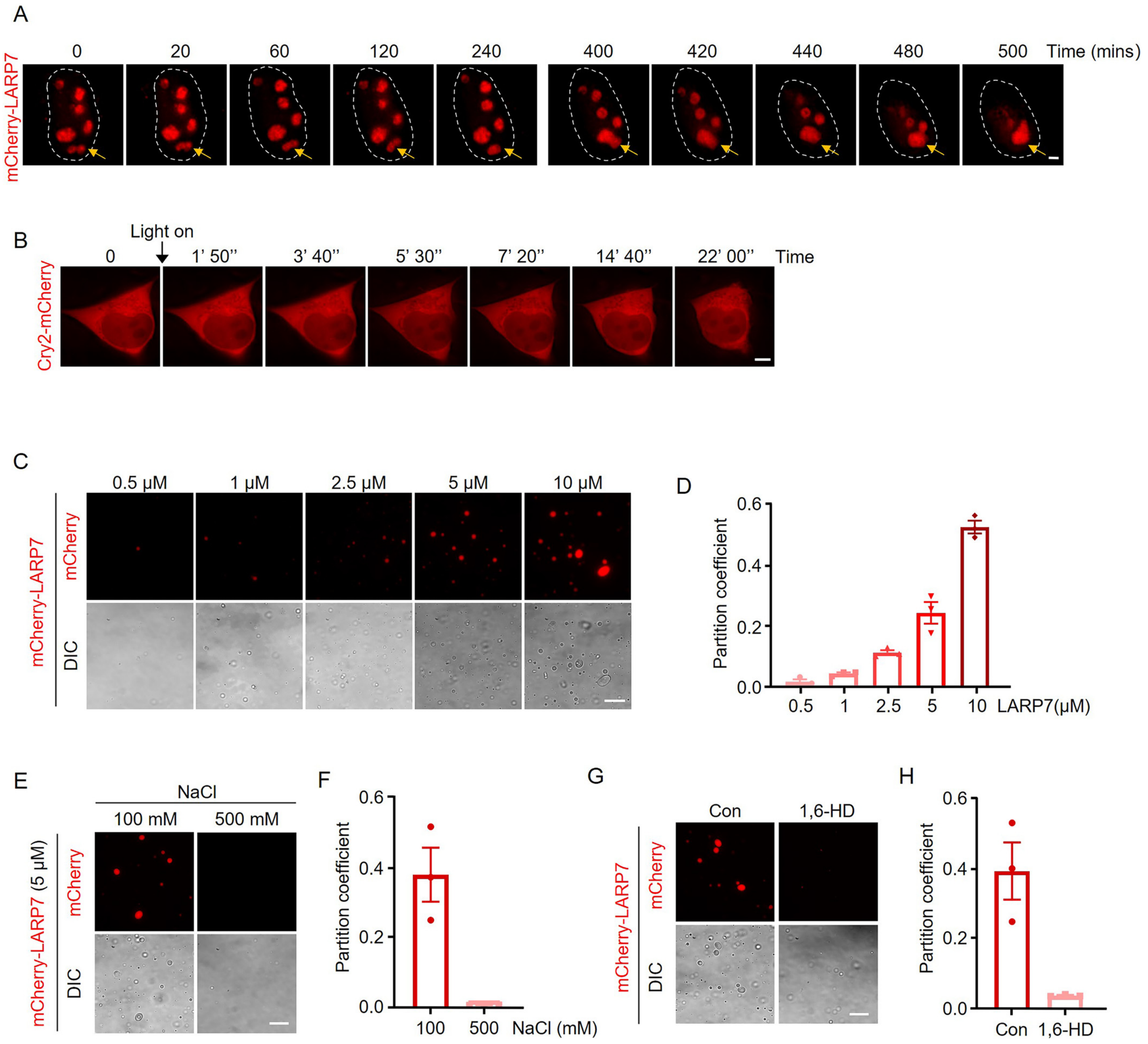

Figure EV2. LARP7 undergoes liquid-liquid phase separation *in cellulo* and in vitro.

(**A**) Timelapse imaging of cells expressing mCherry-LARP7, subjected to laser excitation every 20 min. A droplet fusion event occurs, indicated by the yellow arrows. Scale bar, 5 μm. (**B**) Timelapse imaging of the cell expressing Cry2-mCherry during stimulation by blue light. Scale bar, 5 μm. (**C**) Representative images of mCherry-LARP7 droplets with the indicated concentrations. Scale bar, 20 μm. (**D**) Quantification of partition coefficients for individual conditions in Fig. EV2C. Partition coefficients were calculated as the total fluorescence intensity of droplets divided by the bulk fluorescence intensity of the background. Three random fields were analyzed in each experiment, mean ± SEM. (**E**) Representative images of mCherry-LARP7 droplets (5 μM) with different added concentrations of NaCl. Scale bar, 20 μm. (**F**) Quantification of partition coefficients for individual combinations in Fig. EV2E. Three random fields were analyzed in each experiment, mean ± SEM. (**G**) Representative images of mCherry-LARP7 droplets (5 μM) with 6% 1,6-HD added. Scale bar, 20 μm. (**H**) Quantification of partition coefficients for the experiment in Fig. EV2G. Three random fields were analyzed in each experiment, mean ± SEM.

 © The Author(s)

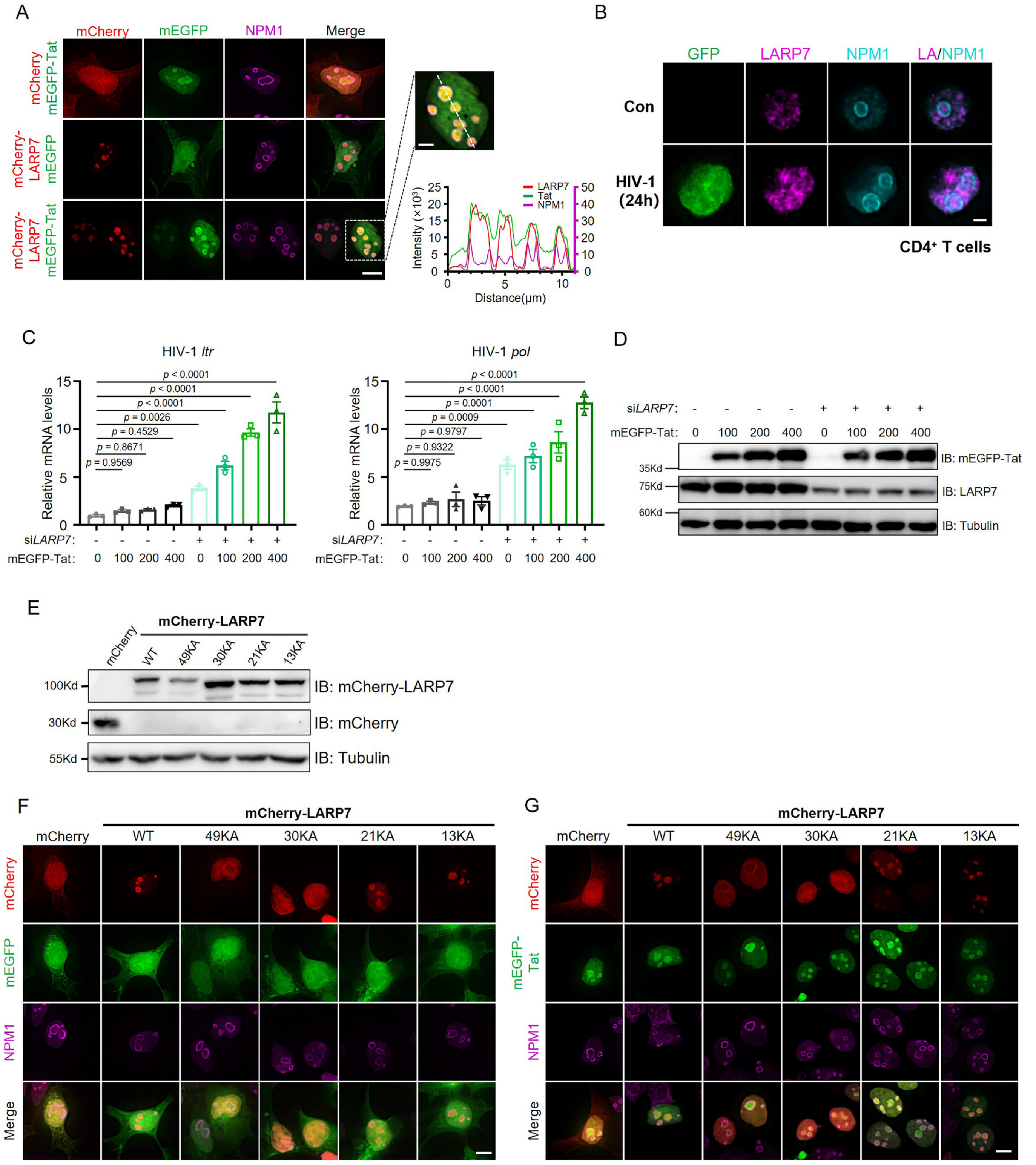
A
mCherry
mEGFP
NPM1
Merge
mCherry mEGFP-Tat
mCherry-LARP7 mEGFP
mCherry-LARP7 mEGFP-Tat
LARP7
Tat
NPM1
Intensity (×10³)
Distance(μm)
B
GFP
LARP7
NPM1
LA/NPM1
Con
HIV-1 (24h)
CD4⁺ T cells
C
HIV-1 ltr
HIV-1 pol
Relative mRNA levels
p < 0.0001
p = 0.0026
p = 0.4529
p = 0.8671
p = 0.9569
p = 0.0001
p = 0.0009
p = 0.9797
p = 0.9322
p = 0.9975
siLARP7:
mEGFP-Tat: 0 100 200 400 0 100 200 400
D
IB: mEGFP-Tat
IB: LARP7
IB: Tubulin
35Kd
75Kd
60Kd
E
mCherry-LARP7
mCherry
WT
49KA
30KA
21KA
13KA
100Kd
30Kd
55Kd
IB: mCherry-LARP7
IB: mCherry
IB: Tubulin
F
G
mEGFP-Tat

Figure EV3. LARP7 forms liquid-liquid phase separation droplets with Tat.

(**A**) Left, representative images of HEK293T cells expressing mCherry-LARP7 and mEGFP-Tat, separately and together. The cells were stained with an anti-NPM1 antibody. Scale bar, 10 µm. Right, the co-localized droplets are enlarged, scale bar, 5 µm. Fluorescence intensity profiles are shown for mCherry-LARP7 (red curve), mEGFP-Tat (green curve) and NPM1 (magenta curve) on the dotted line indicated in the image. (**B**) Immunofluorescence of LARP7 and NPM1 in primary $CD4^+$ T cells 24 h after infection by HIV-1 R7-ΔEnv-GFP. Scale bar, 2 µm. (**C**) RT-qPCR analysis of the mRNA levels of HIV-1 *pol* and *ltr* in individual cells. Each data point represents an independent biological replicate ($n = 3$). One-way analysis of variance (ANOVA) was used for statistical analysis, and exact p values are represented in the figure, mean ± SEM. (**D**) Western blotting analysis of the effect of LARP7 knockdown and the expression of mEGFP-Tat in individual cells in Fig. EV3C. (**E**) Western blotting analysis of mCherry-tagged LARP7 variants expressed in HEK293T cells. (**F**) Representative images of HEK293T cells transfected with mEGFP and mCherry-LARP7 (WT or IDR mutants). Scale bar, 10 µm. (**G**) Representative images of HEK293T cells transfected with mEGFP-Tat and mCherry-LARP7 (WT or IDR mutants). Scale bar, 10 µm.

 © The Author(s)

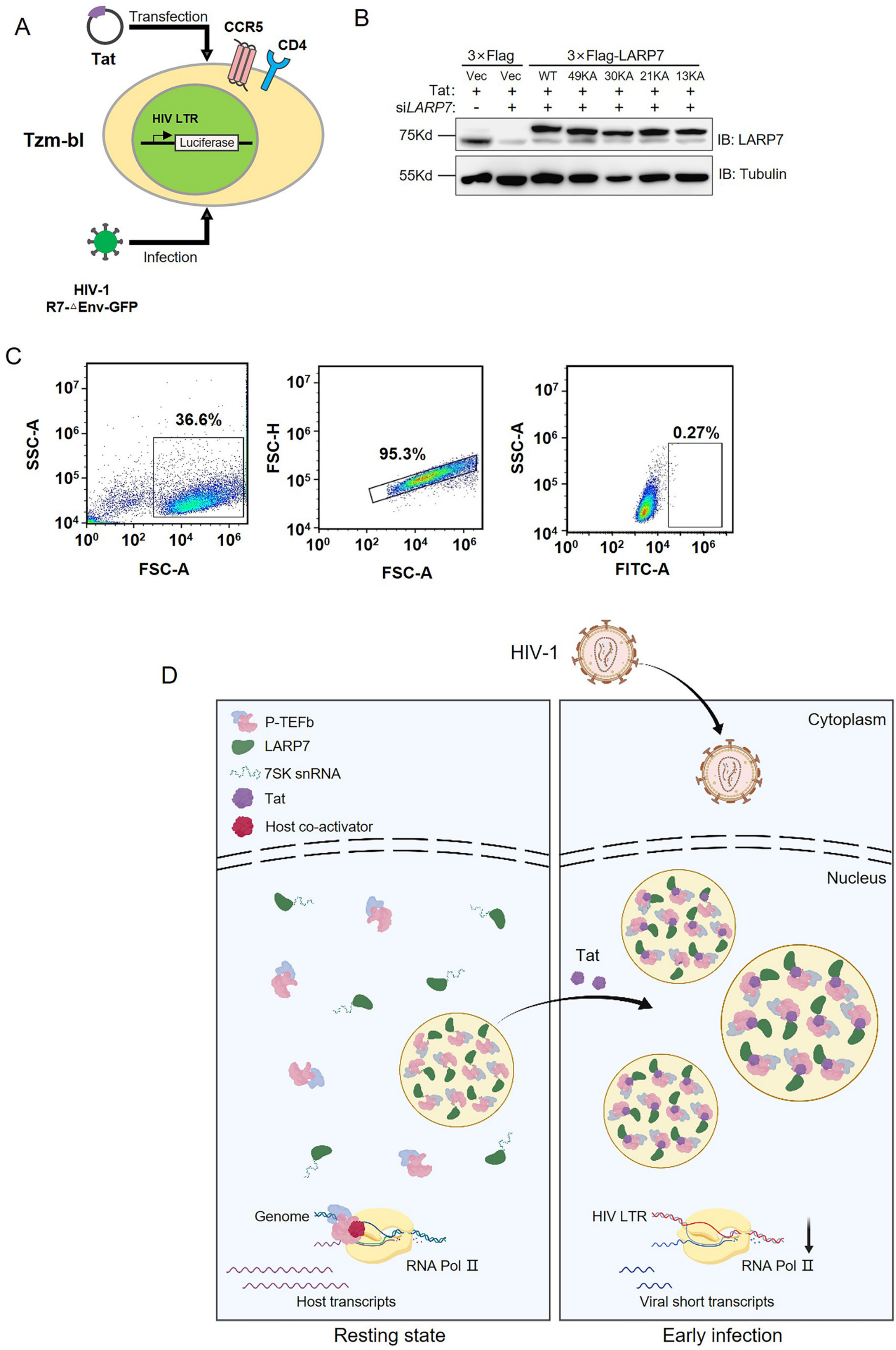
A
Transfection
Tat
CCR5
CD4
Tzm-bl
HIV LTR
Luciferase
Infection
HIV-1
R7-△Env-GFP
B
3×Flag
3×Flag-LARP7
Vec
Vec
WT
49KA
30KA
21KA
13KA
Tat:
si*LARP7*:
75Kd
55Kd
IB: LARP7
IB: Tubulin
C
SSC-A
FSC-A
36.6%
FSC-H
95.3%
SSC-A
FITC-A
0.27%
D
HIV-1
P-TEFb
LARP7
7SK snRNA
Tat
Host co-activator
Cytoplasm
Nucleus
Tat
Genome
RNA Pol Ⅱ
Host transcripts
HIV LTR
RNA Pol Ⅱ
Viral short transcripts
Resting state
Early infection

◀ **Figure EV4. LARP7 inhibits Tat-mediated HIV-1 infection through phase separation.**

(**A**) Schematic illustrating the dual-luciferase reporter assay and HIV-1 infection experiments in the Tzm-bl cell line. (**B**) Western blotting analysis of the effect of LARP7 knockdown and the expression of LARP7 variants in individual cells in Fig. 4A. (**C**) LARP7 was transient knockdown in Tzm-bl cells stably expressing different LARP7 mutants, infected the cells with HIV-1 R7-ΔEnv-GFP, and detected GFP expression 48 h after infection using FACS. (**D**) Model in which LARP7 forms condensates with the 7SK snRNP complex as well as Tat, confining Tat to inhibit HIV-1 transcriptional activation.

 © The Author(s)
